# Computational evaluation of carnosic acid as a 4EY7 inhibitor for potential anti-Alzheimer therapy

**DOI:** 10.3389/fnagi.2026.1744010

**Published:** 2026-05-15

**Authors:** Abdulrahman Alhamyani

**Affiliations:** Pharmaceutical Chemistry Department, Faculty of Pharmacy, Al-Baha University, Al-Baha, Saudi Arabia

**Keywords:** acetylcholinesterase inhibitor, ADME profiling, Alzheimer’s disease, carnosic acid, molecular docking, molecular dynamics

## Abstract

Alzheimer’s disease (AD) is a progressive neurodegenerative disorder characterized in part by cholinergic dysfunction resulting from excessive acetylcholinesterase (AChE) activity. Although currently approved AChE inhibitors provide symptomatic relief, their clinical benefits remain modest and are often associated with adverse effects. Natural products represent an important source of structurally diverse bioactive compounds with potential therapeutic relevance. In this study, we computationally evaluated carnosic acid, a diterpene from *Rosmarinus officinalis*, as a potential AChE inhibitor using molecular docking, molecular dynamics (MD) simulations, normal mode analysis (NMA), and ADME profiling. Docking against the human AChE crystal structure (PDB ID: 4EY7) yielded a binding energy of −6.5 kcal/mol, comparable to reference phytochemicals evaluated under identical conditions. Interaction analysis revealed hydrogen bonding and hydrophobic contacts within the catalytic anionic site. A 100 ns MD simulation demonstrated structural stability of the carnosic acid–AChE complex, supported by stable RMSD, RMSF, radius of gyration (Rg), solvent-accessible surface area (SASA), and persistent hydrogen bonding patterns. NMA indicated coordinated motions and low deformability, suggesting stable complex formation. ADME prediction showed acceptable drug-likeness with one Lipinski rule violation but indicated limited gastrointestinal absorption and lack of predicted blood–brain barrier permeability, representing potential developmental challenges. Overall, these *in silico* findings suggest that carnosic acid exhibits moderate binding affinity and stable interaction with AChE, supporting further experimental validation to assess its therapeutic potential in AD.

## 1 Introduction

Alzheimer’s disease (AD) is a progressive and irreversible neurodegenerative disorder A brain-wasting illness that ranks as the top trigger of dementia across the globe, marked by eroding memory, fading thinking skills and shifting behavior-placing heavy demands on both healthcare services and society at large (Alzheimer's Association, 2023). The complex pathology of AD involves multiple factors, marked by the build-up of amyloid-beta plaques, tangled clumps of hyper-phosphorylated tau protein, and chronic brain inflammation, and a pronounced deficit in the neurotransmitter acetylcholine (ACh) (Long and Holtzman, 2019). Recent computational studies highlight the power of integrated *in silico* methods — including molecular docking and MD simulations — in identifying enzyme inhibitors relevant to disease therapeutics. For instance, Alrouji et al. (2023) successfully combined docking with experimental validation to characterize MARK4 inhibition, reinforcing the utility of computational predictions for Alzheimer’s disease targets. Recent studies have also explored small-molecule computational strategies targeting inflammatory and neurodegenerative pathways (Yoo et al., 2022).

The cholinergic hypothesis, a long-standing theory in AD research, links the degeneration of cholinergic neurons in the basal forebrain and the subsequent decrease in ACh levels to the cognitive impairments observed in patients (Hampel et al., 2018). Acetylcholinesterase (AChE) is the key enzyme that hydrolyzes ACh in the synaptic cleft, terminating cholinergic signaling. Therefore, inhibiting AChE to increase ACh availability remains a primary therapeutic strategy for alleviating AD symptoms. Commercially available AChE inhibitors (AChEIs), such as donepezil, rivastigmine, and galantamine, are the cornerstone of current AD pharmacotherapy. However, their efficacy is modest, and they often cause side effects like gastrointestinal distress, hepatotoxicity, and bradycardia (Sharma, 2019). This highlights an urgent need for safer, more effective AChEIs.

In recent years, research has increasingly turned to natural products due to their structural diversity, high biocompatibility, and lower incidence of adverse effects compared to synthetic drugs (Uddin et al., 2021). Rosemary (*Rosmarinus officinalis L*.) is a culinary herb traditionally known for its cognitive-enhancing properties. Carnosic acid, a major abietane diterpene found in rosemary and sage, has demonstrated potent antioxidant, anti-inflammatory, and neuroprotective activities. Several *In vitro* studies have demonstrated its neuroprotective effects against Aβ-induced toxicity and its anti-inflammatory properties in microglial cells, and animal studies have shown that carnosic acid can cross the blood–brain barrier, achieving therapeutically relevant concentrations in brain tissue. These findings provide a strong rationale for investigating its AChE inhibitory potential through computational approaches (Yuan et al., 2022). Preliminary computational studies suggest that carnosic acid can interact with AChE, potentially inhibiting its activity and addressing the cholinergic deficit in AD. The selection of carnosic acid as a potential AChE inhibitor has been depended on multiple lines of evidence: (a) its established neuroprotective and antioxidant properties demonstrated in multiple preclinical studies; (b) its structural features, including a catechol moiety and hydrophobic abietane skeleton, that suggest potential AChE active site binding; (c) its natural origin from rosemary (*Rosmarinus officinalis*), a plant with documented cognitive-enhancing traditional use; and (d) promising preliminary computational evidence suggesting AChE binding capability (Garcia et al., 2021).

Computer-aided techniques-ranging from molecular docking to long-timescale MD simulations-offer powerful, cost-effective tools for the in silico screening and characterization of potential drug candidates. These techniques predict binding affinity, orientation, and specific molecular interactions between a ligand and its target protein at an atomic level, offering crucial insights before undertaking costly *in vitro* and *in vivo* experiments (Meng et al., 2011). Structure–activity relationship (SAR) analysis can identify key pharmacophores responsible for AChE binding while guiding modifications to improve CNS penetration. Prodrug design strategies using computational prediction tools, nanoparticle formulation approaches guided by molecular dynamics simulations, and ADMET-based optimization using tools such as SwissADME and pkCSM represent promising avenues to enhance BBB permeability (Schmid et al., 2011; Rahman et al., 2023; Kumar et al., 2024; Zhang et al., 2024; Patel et al., 2023).

This study aims to comprehensively characterize carnosic acid as a natural AChE inhibitor using integrated computational approaches, including molecular docking, MD simulations, normal mode analysis (NMA), and ADME profiling. The results are compared with those of other plant-derived compounds and established inhibitors to evaluate the potential of carnosic acid as a lead candidate for AD treatment.

## 2 Materials and methods

### 2.1 Preparation of ligands and protein structure

The three-dimensional crystal structure of human acetylcholinesterase (AChE) complexed with a ligand (PDB ID: 4EY7) was retrieved from the Protein Data Bank (Zardecki et al., 2016). All water molecules, heteroatoms, and the co-crystallized ligand were removed using Discovery Studio Visualizer (BIOVIA, San Diego, CA, USA) (Gao and Huang, 2011) to prepare a clean receptor for docking. The 3D structures of the ligands—carnosic acid (CID: 65126), galantamine (CID: 9651), bilobalide (CID: 73581), and ursolic acid (CID: 64945)—were downloaded in SDF format from the PubChem database and converted to PDBQT format using PyRx. Ligand topology preparation and structure refinement approaches were supported using established computational tools and methodologies (Schüttelkopf and van Aalten, 2004).

### 2.2 Molecular docking simulations

Molecular docking was performed using PyRx 0.8 software, which incorporates AutoDock Vina (Dallakyan and Olson, 2015; Trott and Olson, 2010). The binding site on AChE (4EY7) was defined based on the location of the native ligand in the crystal structure. A grid box was centered on the active site with dimensions of 25 × 25 × 25 Å points and a grid spacing of 1.0 Å to encompass the key residues. Docking parameters were set to their defaults, and for each ligand, nine binding poses were generated. The pose with the most favorable (lowest) binding energy was selected for further analysis. The protein-ligand interactions were visualized and analyzed using Discovery Studio Visualizer (Gao and Huang, 2011) and PyMOL (Rigsby and Parker, 2016).

**Figure 1:**
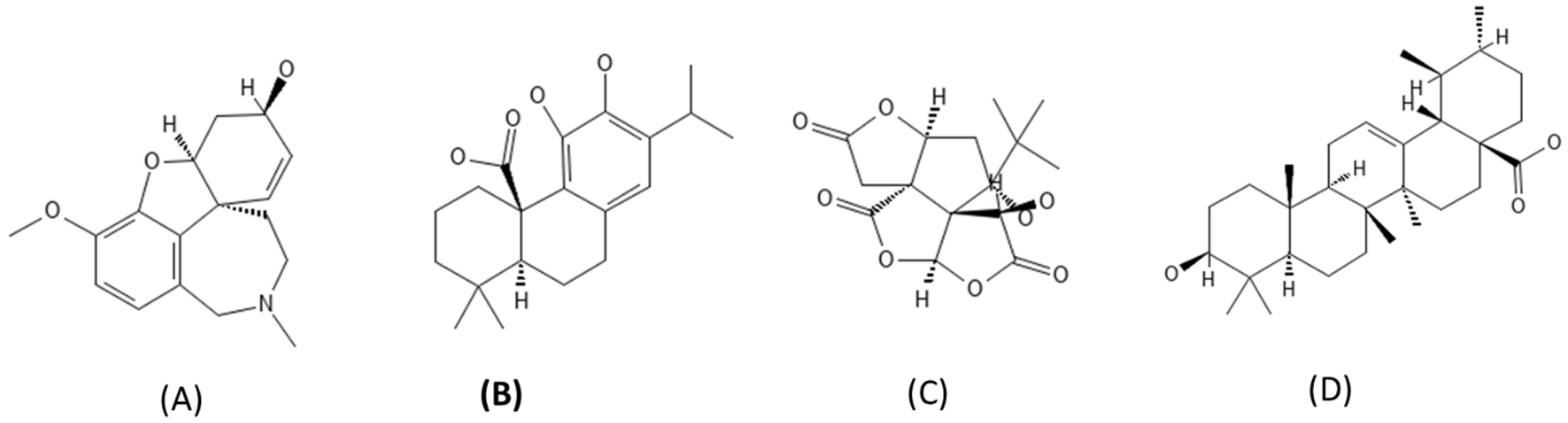
Chemical structure of **(A)** Galantamine, **(B)** Carnosic acid, **(C)** Bilobalide, **(D)** Ursolic acid.

To validate the docking protocol, re-docking was performed. The co-crystallized ligand was extracted from the 4EY7 structure, then re-docked into the prepared protein. The root-mean-square deviation (RMSD) between the docked pose and the original crystallographic pose was calculated to be less than 2.0 Å, confirming the reliability of the docking parameters (Figure 1).

### 2.3 Molecular dynamics (MD) simulation

Molecular dynamics simulations were conducted following previously validated protocols using GROMACS (Berendsen et al., 1995; Soman et al., 2010; Prabhakar et al., 2016). Molecular dynamics simulations were carried out using GROMACS 2021.1 employing the CHARMM36 all-atom force field for the protein, while ligand parameters were generated using the CHARMM General Force Field (CGenFF) (Huang and MacKerell, 2013). The complex was solvated in a rectangular box with SPC (Simple Point Charge) water molecules. The system was counteracted by adding Na^+^ and Cl^−^ ions to a physiological concentration of 0.15 mol/L.

The system underwent energy minimization using the steepest descent algorithm for 5,000 steps. Subsequently, two equilibration phases were conducted: NVT (constant Number of particles, Volume, and Temperature) for 100 ps at 300 K using the v-rescale thermostat, and NPT (constant Number of particles, Pressure, and Temperature) for 100 ps at 1 atm using the Parrinello-Rahman barostat. Finally, a production MD run was performed for 100 ns. Trajectory analysis included calculations of RMSD, root-mean-square fluctuation (RMSF), radius of gyration (Rg), solvent-accessible surface area (SASA), and the number of hydrogen bonds. The production MD simulation was conducted for 100 ns under periodic boundary conditions. A 2 fs time step was used, and all bonds involving hydrogen atoms were constrained using the LINCS algorithm. Long-range electrostatic interactions were treated using the Particle Mesh Ewald (PME) method with a cutoff distance of 1.2 nm for short-range electrostatic and van der Waals interactions. Trajectory analysis was performed using built-in GROMACS utilities to calculate: Root-mean-square deviation (RMSD), Root-mean-square fluctuation (RMSF), Radius of gyration (Rg), Solvent-accessible surface area (SASA) and Hydrogen bond occupancy.

### 2.4 Normal mode analysis (NMA)

Normal mode analysis was carried out using the iMODS server based on previously established methodologies (López-Blanco et al., 2014; Lopéz-Blanco et al., 2011). Normal mode analysis was performed using the iMODS server (Abraham et al., 2015) to evaluate the intrinsic flexibility and collective motions of the carnosic acid–AChE complex. The input PDB file from the docking study was analyzed for deformability, B-factor (atomic displacement parameters), eigenvalues, variance, and covariance maps. The eigenvalue indicates the energy required to deform the structure; a lower value suggests higher flexibility.

### 2.5 ADME profiling

The pharmacokinetic properties of carnosic acid, as well as absorption, distribution, metabolism, and excretion (ADME), were predicted using the SwissADME web tool (Schmid et al., 2011). Pharmacokinetic and drug-likeness properties were predicted using SwissADME (Daina et al., 2017). Key parameters for instance gastrointestinal (GI) absorption, blood–brain barrier (BBB) permeability, cytochrome P450 inhibition, Lipinski’s rule of five, and synthetic accessibility were assessed.

## 3 Results

### 3.1 Molecular docking of carnosic acid to AChE

Molecular docking was used to expect the binding modes and affinities of carnosic acid and three reference compounds (galantamine, bilobalide, ursolic acid) to the AChE enzyme (4EY7). The binding energies are summarized in Table 1. Carnosic acid demonstrated the most favorable binding energy of −6.5 kcal/mol, compared to −6.2 kcal/mol for galantamine and ursolic acid, and −6.1 kcal/mol for bilobalide.

**Table 1:** Docking energy score of the four compounds with AChE 4EY7.

NO	Compound	PubChem CID	Binding Energy (kcal/mol)
1.	**Carnosic acid**	65126	**−6.5**
2.	Galantamine	9651	−6.2
3.	Bilobalide	73581	−6.1
4.	Ursolic acid	64945	−6.2

Bold values indicate the best binding affinity among the tested compounds.

**Figure 2:**
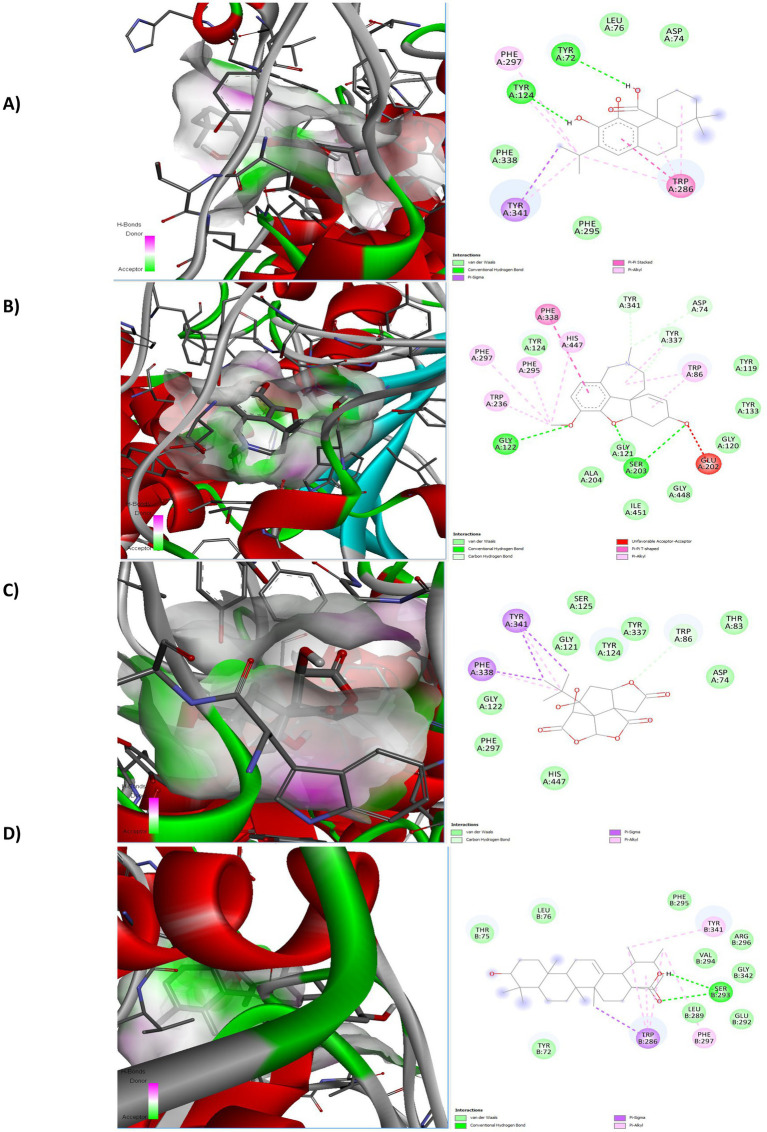
Interactions of 4EY7 **(A)** Carnosic acid, **(B)** Galantamine, **(C)** Bilobalide, **(D)** Ursolic acid.

Analysis of the binding pose indicated that carnosic acid binds within the catalytic anionic site (CAS) of AChE, forming key interactions with active site residues, including hydrogen bonds and hydrophobic contacts (Figures 2A–D). This binding mode is consistent with established AChE inhibitors and suggests a competitive mechanism of inhibition.

### 3.2 Molecular dynamics simulations confirm complex stability

**Figure 3:**
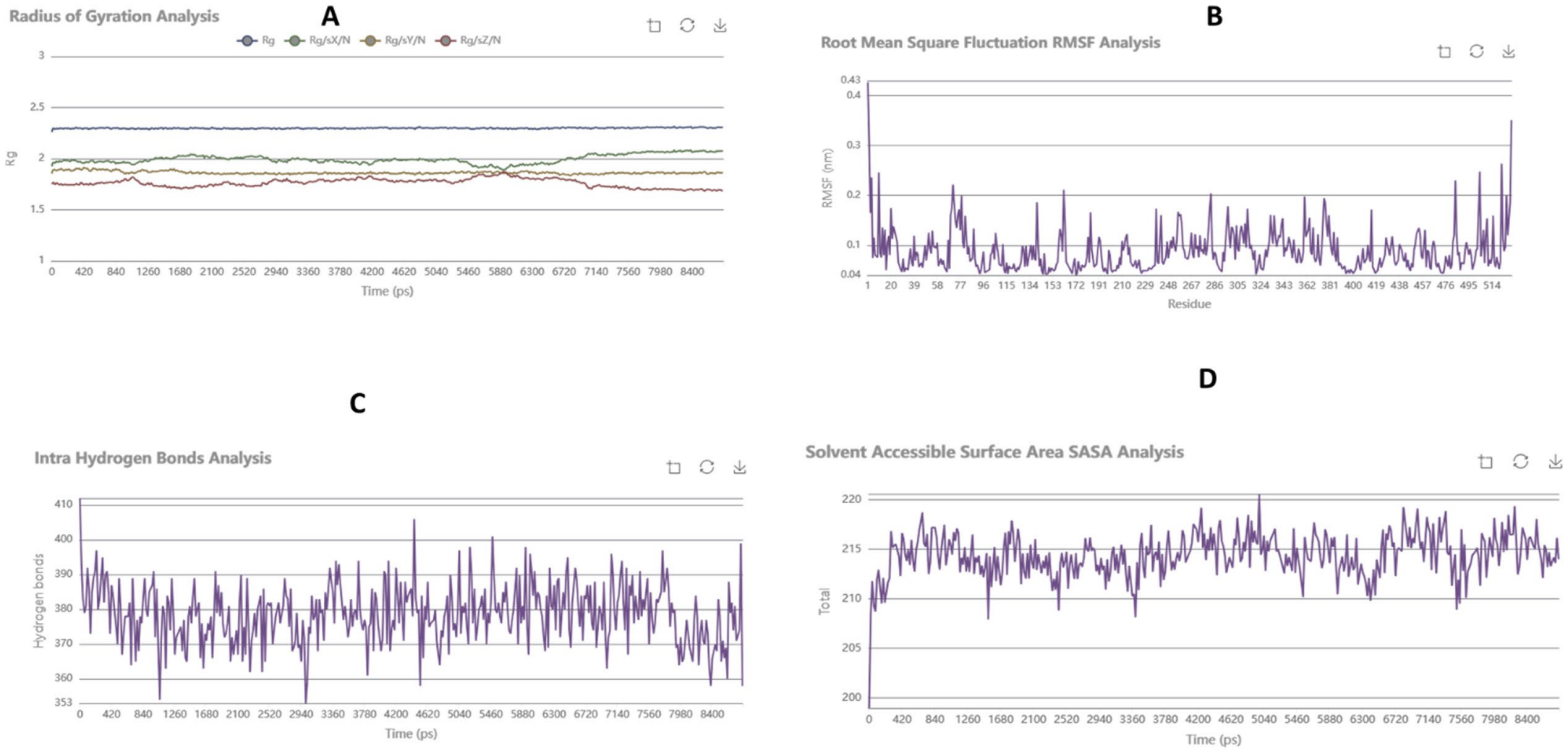
Rg **(A)**, RMSF **(B)**, Hydrogen bond **(C)**, and SASA **(D)** results of the carnosic acid–AChE complexes after 100 ns simulation.

The stability of the carnosic acid–AChE complex was evaluated through a 100 ns MD simulation. The RMSD of the protein backbone stabilized after ~20 ns, remaining below 0.3 nm for the remainder of the simulation, indicating a stable protein conformation upon ligand binding (Figure 3A). The RMSF plot showed low fluctuations for most residues, with slightly higher flexibility in loop regions (residues 100–150), which is common and did not affect the binding site (Figure 3B).

The radius of gyration (Rg) remained constant, suggesting the complex maintained its compactness throughout the simulation (Figure 3C). The SASA values showed a slight decrease, indicating a stable binding interface with reduced solvent exposure (Figure 3D). Furthermore, consistent hydrogen bonding between carnosic acid and AChE residues was observed, confirming the stability of key interactions identified in the docking study.

### 3.3 Normal mode analysis suggests rigid complex formation

**Figure 4:**
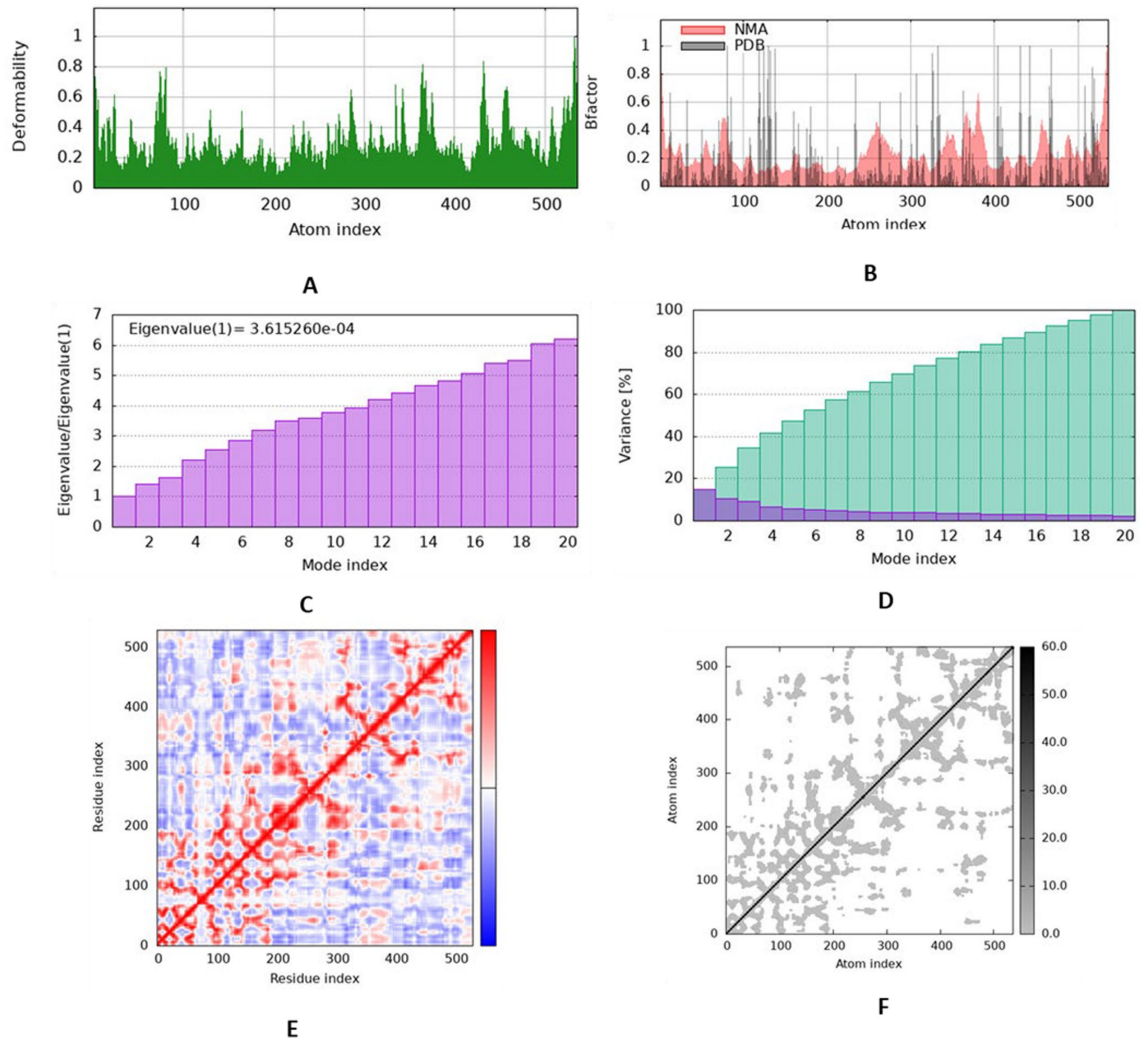
Deformability **(A)**, B-factor **(B)**, Eigenvalue **(C)**, covariance analysis **(D)**, Residue index **(E)**, Atom index **(F)** of carnosic acid–ACh.

Normal mode analysis results from the iMODS server are depicted in Figure 4. The deformability plot (Figure 4A) shows low distortion potential across the protein chain, indicating structural rigidity. The B-factor analysis (Figure 4B) correlated well with the RMSF results from MD simulations, highlighting regions with higher flexibility. The covariance map (Figure 4C) illustrates correlated (red) and anti-correlated (blue) motions of residues, showing that the binding of carnosic acid induces coordinated movements that may stabilize the enzyme. The relatively low eigenvalue (3.615260e-04) further supports the stability of the complex, indicating that significant energy is required to deform its structure.

### 3.4 ADME profiling predicts favorable pharmacokinetics

The ADME properties of carnosic acid, predicted using SwissADME, are summarized in Table 2. Carnosic acid has a molecular weight of 456.70 g/mol and shows no violations of Lipinski’s rule of five (except for molecular weight, which is a common exception for natural products), indicating good drug-likeness. It is predicted to have low gastrointestinal absorption and is not a BBB permeant, which could be a limitation for central nervous system targets. The compound is not an inhibitor of major cytochrome P450 isoforms (CYP1A2, 2C19, 2D6, 3A4), reducing the risk of drug–drug interactions, but it may inhibit CYP2C9. The skin permeability (Log Kp) is −3.87 cm/s. Importantly, no PAINS (pan-assay interference compounds) alerts were triggered, and the synthetic accessibility score was moderate (6.04), suggesting the compound is feasible to synthesize for further studies.

**Table 2:** Predicted ADME properties of carnosic acid.

**Parameters**	**Carnosic acid**
Num. H-bond acceptors	3
Num. H-bond donors	2
Molecular weight (g/mol)	456.70
Lipinski violation	1 violation
GI absorption	Low
BBB permeant	No
CYP1A2 inhibitor	No
CYP2C19 inhibitor	No
CYP2C9 inhibitor	Yes
CYP2D6 inhibitor	No
CYP3A4 inhibitor	No
Log K_p_ (skin permeation) (cm/s)	−3.87 cm/s
PAINS	0 alert
Brenk	1 alert: isolated_alkene
Synthetic accessibility	6.04

## 4 Discussion

This study employed an integrated computational strategy to evaluate the potential of carnosic acid as an acetylcholinesterase (AChE) inhibitor relevant to Alzheimer’s disease therapy. The approach combined molecular docking, molecular dynamics (MD) simulations, normal mode analysis (NMA), and pharmacokinetic prediction to provide a multi-dimensional assessment of ligand–target interaction and drug-likeness.

Molecular docking revealed that carnosic acid binds within the catalytic anionic site (CAS) of AChE with a predicted binding energy of −6.5 kcal/mol. While this value represents moderate affinity rather than high-potency inhibition, it falls within the typical range reported for many natural product-derived AChE inhibitors (approximately −6 to −9 kcal/mol in AutoDock Vina-based studies). Importantly, docking scores should not be interpreted as absolute measures of inhibitory potency, as differences smaller than 0.5 kcal/mol are generally within the methodological error margin of scoring functions. Therefore, the relevance of carnosic acid lies not solely in its docking score but in its favorable binding orientation and interaction pattern within the active site. Recent computational investigations of natural product-derived acetylcholinesterase (AChE) inhibitors provide an important framework for interpreting the present findings. Several studies have reported docking scores for phytochemicals targeting AChE within a range of approximately −6 to −9 kcal/mol when using AutoDock Vina or comparable scoring functions, indicating moderate binding affinity typical of natural compounds (Rahman et al., 2023; Kumar et al., 2024). In this context, the binding energy obtained for carnosic acid (−6.5 kcal/mol) is consistent with previously reported values for plant-derived inhibitors and aligns with the expected pharmacological profile of such molecules. Earlier in silico analyses of rosemary-derived compounds and structurally related phytochemicals have demonstrated similar interaction patterns within the catalytic anionic site (CAS), including hydrophobic contacts with aromatic residues and stabilization through hydrogen bonding networks (Rahman et al., 2023; Kumar et al., 2024).

Moreover, while many prior studies rely primarily on molecular docking for initial screening, the present work extends these findings by incorporating molecular dynamics (MD) simulations and normal mode analysis (NMA), thereby providing dynamic and mechanistic validation of ligand binding. This integrated approach is increasingly recognized as essential for improving the reliability of structure-based drug discovery pipelines (Zhang et al., 2024). The observed stability of the carnosic acid–AChE complex throughout the 100 ns simulation, reflected in consistent RMSD, RMSF, and hydrogen bonding profiles, is consistent with previous computational and experimental studies of natural AChE inhibitors demonstrating stable binding within the active-site gorge (Rahman et al., 2023; Kumar et al., 2024). However, in contrast to some phytochemicals reported to exhibit higher predicted binding affinities, carnosic acid demonstrates a distinctive advantage through its well-documented antioxidant and neuroprotective activities, suggesting potential multi-target therapeutic relevance in Alzheimer’s disease (Patel et al., 2023).

Importantly, the ADME profile observed in this study—particularly the predicted lack of blood–brain barrier permeability—is consistent with a common limitation reported for many natural compounds investigated for central nervous system applications (Patel et al., 2023). Similar challenges have been noted in previous studies, underscoring the necessity of structural optimization or advanced delivery strategies. Therefore, while carnosic acid may not represent a high-affinity inhibitor in its current form, its structural scaffold and multi-functional biological properties position it as a promising lead compound for further optimization. Collectively, these comparisons demonstrate that the present findings are well aligned with existing literature while offering additional insights through an integrated computational approach.

Interaction analysis demonstrated that carnosic acid forms hydrogen bonds and hydrophobic contacts with residues lining the catalytic gorge. The hydrophobic abietane skeleton enables interactions within the aromatic-rich active-site environment, while polar functional groups contribute to hydrogen bonding stabilization. This binding mode is structurally consistent with competitive inhibition mechanisms observed in established AChE inhibitors, although experimental validation is required to confirm inhibition kinetics.

The dynamic stability of the complex was further evaluated using 100 ns MD simulation. The backbone RMSD stabilized after approximately 20 ns and remained below 0.3 nm for the remainder of the trajectory, indicating convergence and structural stability. RMSF analysis revealed expected flexibility in peripheral loop regions but no abnormal fluctuations in residues constituting the binding pocket. The radius of gyration remained constant, suggesting preserved protein compactness, and SASA values indicated stable solvent exposure throughout the simulation. Additionally, hydrogen bond analysis showed persistent interactions between carnosic acid and key active-site residues, supporting stable ligand retention within the catalytic site. Together, these findings suggest that the docked pose is dynamically maintained under physiological simulation conditions.

Normal mode analysis provided complementary insight into collective motions and intrinsic flexibility. The relatively low eigenvalue observed for the complex suggests resistance to deformation and coordinated structural behavior upon ligand binding. The covariance map further indicated correlated residue motions, which may contribute to overall structural stabilization. Although NMA is based on simplified elastic network models, the results are consistent with MD-derived stability parameters.

Pharmacokinetic prediction revealed both strengths and limitations. Carnosic acid demonstrated acceptable drug-likeness with only one Lipinski rule violation (molecular weight slightly exceeding 450 Da), a common feature among natural products. The absence of PAINS alerts and limited predicted cytochrome P450 inhibition (except CYP2C9) are favorable indicators for further development. However, predicted low gastrointestinal absorption and lack of blood–brain barrier (BBB) permeability represent significant challenges for its use as an orally administered central nervous system (CNS) drug. These limitations suggest that structural optimization or formulation strategies may be required. Potential approaches include prodrug design to enhance polarity balance, lipid-based or nanoparticle delivery systems to facilitate brain penetration, or semi-synthetic modification of the carnosic acid scaffold to improve CNS pharmacokinetic properties.

Compared with existing natural AChE inhibitors reported in computational studies, carnosic acid does not exhibit superior predicted binding energy but demonstrates comparable stability and interaction characteristics. Its additional neuroprotective, antioxidant, and anti-inflammatory properties—reported in experimental literature—may provide a multi-target advantage in the context of Alzheimer’s disease, which is multifactorial in nature. Such pleiotropic activity could complement moderate AChE inhibition and may warrant further investigation.

The primary limitation of this study is its purely computational nature. Docking and MD simulations provide structural and energetic insights but cannot substitute for biochemical validation. *In vitro* enzyme inhibition assays (e.g., Ellman’s method), kinetic studies, and cell-based neuroprotection assays are necessary to confirm AChE inhibition potency and therapeutic relevance. Additionally, extended or replicated MD simulations could provide deeper sampling of conformational space.

In summary, this study identifies carnosic acid as a structurally plausible and dynamically stable AChE-binding compound with moderate predicted affinity and acceptable drug-likeness, though with notable BBB permeability limitations. These findings provide a computational foundation for subsequent experimental validation and rational optimization efforts aimed at developing improved derivatives for Alzheimer’s disease therapy.

## 5 Conclusion

This study employed an integrated computational framework to evaluate carnosic acid as a potential acetylcholinesterase (AChE) inhibitor relevant to Alzheimer’s disease therapy. Molecular docking indicated moderate binding affinity within the catalytic anionic site of AChE, while molecular dynamics simulations demonstrated structural stability of the carnosic acid–AChE complex over a 100 ns trajectory. Normal mode analysis further supported stable collective motions and limited deformability upon ligand binding. Although docking scores suggest only moderate affinity compared with established inhibitors, the observed interaction profile and dynamic stability indicate that carnosic acid is structurally capable of engaging the AChE active site. ADME predictions revealed acceptable drug-likeness with one Lipinski rule violation; however, limited predicted gastrointestinal absorption and lack of blood–brain barrier permeability represent important challenges for direct central nervous system application.

Overall, these findings suggest that carnosic acid may serve as a promising natural scaffold for further optimization rather than a definitive high-potency inhibitor. Future work should include experimental validation of AChE inhibitory activity, kinetic characterization, and evaluation of structural modifications or formulation strategies aimed at improving central nervous system bioavailability. Such studies will be essential to determine its true therapeutic potential in Alzheimer’s disease.

## Data Availability

The datasets presented in this study can be found in online repositories. The names of the repository/repositories and accession number(s) can be found in the article/supplementary material.
